# Out of Evil, Good

**Published:** 1876-06

**Authors:** Celia Logan


### *OUT OF EVIL, GOOD.*

BY CELIA LOGAN.

THAT a woman should be directly, or indirectly, the cause of a great man's fall, must be a matter of poignant regret to every woman who thinks and feels; for Belknap was a great man, if not on account of personal distinction, then because of the exalted position which he held, in consequence of which his wife was a great lady, and the story of the ruin caused by her inordinate love of self-adornment and display is condemned and deplored in every household in the land; but if Mrs. Belknap's course and its disastrous results should bear a lesson and a warning to American women, then out of evil will come good.

If reform in the matter of extravagance in dress should be brought about by her evil example, then will there be some compensation for "the nation's shame."

Extravagance in dress, and love of luxury generally, is universal in the United States, especially among the weaker sex, and are carried to such a pitch that we needed, actually needed, some such terrible fate as Mrs. Belknap's to alarm and shock our women into reforming altogether.

Would we could point to her as a solitary instance of this mad fever for costly attire to be obtained by fair means or foul, but we have no such solace; on the contrary, it is not too much to say that, with the exception of our richest ladies, every woman in the length and breadth of the land dresses beyond her means.

Mrs. Belknap's is not the only case where the ruin of the husband has been completed by the remorseless, unwarranted, unnecessary, and sinful extravagance of the wife; it only stands out more strikingly than in ordinary cases, appearing more wanton and wicked from the high position of the couple, and her exceptional opportunities for rushing headlong from peculation to disgrace.

Instead of moaning over, or condemning, or sympathizing with her, would it not be a good idea to consider her simply as a bad example, and discuss her career only to avoid the weakness and unprincipled vanity which has led to her downfall?

If this soul-sickening disgrace at Washington could but inaugurate an universal dress reform movement, then we shall not have suffered from it entirely in vain. I do not mean a reform movement to transform petticoats into pantaloons, but to substitute calico for silks, and pinchbeck for diamonds, if a woman's husband or father can afford no better; but in order to accomplish this, we must remove the prejudice which generally exists against non-stylish dressing, and the idea that many men have, that they must dress their ladies magnificently, in order that they as business men shall appear to be flourishing.

Instead of battling so bitterly over what of women's work shall be admitted to the Centennial, would not the noblest work of the American woman be the exhibition of herself in the streets, or elsewhere, in robes of native manufacture and home make?

The women of the Revolution, as a rule, wore plain dresses of inexpensive material. Is not the Centennial a most appropriate year to revert to the simplicity and economy of our ancestresses? During the Rebellion, the ladies of the South showed us what self-denial in the matter of personal adornment our sex is capable of, and that it is quite possible to dress in non-imported fabrics. This great dress reform movement, which current events seem to suggest, is not like universal suffrage, depending on man to permit; it lies solely with our sex, and by us alone can be accomplished.

By such a sweeping reformation in dress, woman would gain man's respect and gratitude, and wring from him the acknowledgement of her capability and self-government.

But for Mrs. Belknap's extortionate demand upon him for fine raiment and magnificent living, the late Secretary of War might never have been tempted to swerve from the strict confines of integrity, and it behooves every woman to take into serious consideration the grave question of extravagance in dress, which bids fair to plunge the country into ruin. In the days of Martha Waghington, women were but too happy to sacrifice all for our country, but now our country it would seem, must be sacrificed for women.

Let us suppose, however, what is possible, that the majority of women of the United States would be glad, and hail it as a relief, not to be obliged to spend so much money for mere clothing, because for every lady to dress as well as every other lady, no matter what the difference in their circumstances may be, is a terrible tax on the poorer one, and too often, as in Mrs. Belknap's case, engenders envy, and begets, if not absolute vice, a laxity of moral principle, which in its turn undermines the probity of others.

Nevertheless, despite all argument, women will follow the fashion, and will even wear alpaca instead of silk, if it be the mode; therefore, if the leaders of society in every city were to make it a matter of duty to influence others to dress plainly by their example, a general fashion would thus be set, and the reform easily brought about, and that it might be the more speedily and effectively accomplished, it would be excellent if ladies would form dress committees. As crusaders and other reformers, our sex has done good work, and if they were to take up the matter under consideration in earnest, within six months' time no reproach could be cast on American women on the score of unwarranted extravagance in dress.

What a boon it would be if every lady in the United States would pledge herself to buy none but American silks! What an impetus it would give to home manufacture! In a few years our silks would doubtless equal the imported, and we should not only rid ourselves of much import duty, but would create a great industry for our native land! Truly, foreign countries have had enough of our money, it is time that our own people should benefit by it.

It would be well to take, what would cost us nothing, a few lessons in domestic economy from England, where no one thinks it beneath his dignity to live within his income.

English women do not possess what we consider taste in dress, but they dress sensibly, comfortably, and inexpensively.

While there is no actual livery to mark class differences, there are strong, unwritten sumptuary laws in England still. The female servant is not permitted, on pain of losing her place, to wear any material more expensive than calico, or, as they call it, print; she must neither wear ribbons, nor flowers, nor flounces, nor any finery whatever. Silks, satins, velvets, laces, feathers and diamonds are left almost entirely to the nobility and gentry, with whom the lower classes do not attempt to vie. To be dressed beyond her station in life is no credit, but a reproach to an English woman.

Another admirable thing is, that the style of dress to be worn on every occasion is settled beforehand, a sort of regulation suit, and as the fashions do not change as rapidly as with us—for they are the last women to adopt a fashion, and the last to resign it—ladies are enabled to wear their clothes out, without feeling that they look old-fashioned, nor have they any false pride about being seen repeatedly in the same dresses.

As a sample of the "regulation dress" take the fact that in every theater, in every place of amusement, every evening, every lady will wear a white wreath, white kid gloves, with a red opera cloak thrown over her bare shoulders; and at every ceremonious dinner party, reception, or ball, fashion insists that the lady at sixty, as well as the lass of sixteen, shall wear a waist cut low in the neck, which reminds me of the gentleman who sat between two ladies, and being called upon to make an after-dinner speech, made a most courteous allusion to his bosom friend on the right, and his still more bosom friend on the left.

The English are a methodical, steady, home-loving people. They systematize their lives, and have a purpose in them—that purpose is usefulness. To this end, they spare no pains in the education of their children. In order to teach them the value and proper outlay of money, in well-to-do families, every little one is given a trifle, say a shilling or sixpence a week to spend as it pleases, upon the condition that it should render an account correctly balanced.

It is pleasant to see children flocking to their parents, when the day of reckoning comes, each boy and girl with a little account-book, hoping that its expenditures may be approved of. The system of an allowance begins with the mistress of the house. She is given so much for personal, so much for domestic use. The usual allowance for a young girl of the middle class is twenty pounds a year, not more than enough in this country to buy a girl a silk dress, and from forty to fifty pounds yearly for the upper classes.

Out of these twenty or fifty pounds a young lady must not only clothe herself from head to foot throughout the four seasons, but she must give to the poor and to the Church; in short, this small sum must include all her expenses except board. No young girl in the middle classes could dress on that money here, but clothing and everything else is cheaper than with us; besides, English people are deadly enemies to waste, have reduced economy in small matters to a science, and are in nowise ashamed to be careful of the pence, but they give very freely to the poor, and there is no country in the world where so many charitable institutions are supported by voluntary contributions.

Every mother of a family instructs her daughters in the knowledge of housekeeping; according to their age they take their turn in managing the house, the eldest daughter one week, the second the next, so that when girls marry, they are thoroughly capable of directing servants, and have none of the bitter experience of our girls when they begin housekeeping for themselves.

Young people are not afraid of marrying on small incomes, for the man knows that the woman will live within his means. They do not begin life in boarding-houses, but draw apart from the world, and found neat, retired little houses of their own.

Girls are not thrown freely into the society of gentlemen. The English do not contract marriage as hastily as we do, but approve of long courtships, during which they have time to become acquainted with each other's character, consequently they bestow and receive much happiness in wedded life.

Men have confidence in, and respect for their women, who make exemplary wives and mothers. Owing to their very careful bringing up, and their commendable plan of living strictly within their means, English women do not lose their head, nor become dazzled as readily as American women, when elevated to brilliant positions, and surrounded by unaccustomed luxury and wealth. Our stock is however, as good as theirs, though our home training lacks their uprightness and completeness.

While theirs is an old country, ours has hardly passed its infancy- May such national sorrows as have lately fallen on us, chasten us, and out of the evil of one lady's weakness and retributive wretchedness, come some good to the now humiliated daughters of the Republic.

